# Parietal Cortex Signals Come Unstuck in Time

**DOI:** 10.1371/journal.pbio.1001414

**Published:** 2012-10-30

**Authors:** Erik P. Cook, Christopher C. Pack

**Affiliations:** 1 Department of Physiology, McGill University, Montreal, Quebec, Canada; 2 Neurology & Neurosurgery, McGill University, Montreal, Quebec, Canada

## Abstract

Humans and other animals are surprisingly adept at estimating the duration of temporal intervals, even without the use of watches and clocks. This ability is typically studied in the lab by asking observers to indicate their estimate of the time between two external sensory events. The results of such studies confirm that humans can accurately estimate durations on a variety of time scales. Although many brain areas are thought to contribute to the representation of elapsed time, recent neurophysiological studies have linked the parietal cortex in particular to the perception of sub-second time intervals. In this Primer, we describe previous work on parietal cortex and time perception, and we highlight the findings of a study published in this issue of *PLOS Biology*, in which Schneider and Ghose [1] characterize single-neuron responses during performance of a novel “Temporal Production” task. During temporal production, the observer must track the passage of time without anticipating any external sensory event, and it appears that the parietal cortex may use a unique strategy to support this type of measurement.

## The Neural Basis of Time Perception

Neuroscientists and psychologists have long been interested in time perception, as accurate timing serves as the basis for many behaviors, ranging from foraging strategies in birds [2] to skilled musical performances. Although there are a variety of types of time perception, we will focus here on the perception of duration, which William James characterized as the perception of “empty time flow” [3]. James, in an 1886 article in the *Journal of Speculative Philosophy*, proposed that time perception might be similar to the perception of other quantities, particularly those related to physical space. Speculative as the idea might have been, it already had some support from the observation that participants' ability to discriminate durations was similar to their ability to discriminate other physical quantities, such as brightness or sound intensity. In all cases, people turned out to be better at distinguishing between small quantities (durations, intensities, etc.) than between large ones.

Critical biological functions require knowledge of absolute temporal intervals at multiple scales, from milliseconds used to coordinate precise motor commands to hours used to regulate the sleep-wake cycle. It has long been held that computing time intervals, even at multiple scales, requires at least one clock and a subsequent accumulator to count the clock-ticks [4]. For example, computers have a single fast clock that is accumulated at different rates to compute time from microseconds to days. By comparison, evolution seems to have produced multiple neural clocks using a variety of physiological mechanisms. Unlike a single, functionally organized system on which neuroscientists can focus their research efforts, there appears to exist multiple, diffuse, and interconnected timing systems in the brain that represent different absolute scales [5].

Regions of the thalamus, striatum, and cortex (especially posterior parietal and prefrontal areas) have all been implicated in a variety of timing tasks. For example, studies have shown that parietal cortex [6], basal ganglia [7], cortico-striatal loops [8], cerebellum [9], and prefrontal cortex [10] have activity correlated with the perception of time. Mechanisms such as synaptic plasticity, neural adaptation, and neural circuit dynamics [11] have been shown to be sufficient to allow neural networks to modulate their activity as a function of duration [5].

The many brain areas and proposed biophysical mechanisms that would allow neural circuits to represent different time scales are both a fascinating and challenging neuroscience topic. Although early studies on patients suffering from Parkinson's disease [12] or cerebellar dysmetria [13] suggested that the basal ganglia and/or the cerebellum might be responsible for time perception, there was always the possibility that these results were influenced by more general sensory, motor, or cognitive factors [14]. We refer the reader to several excellent recent reviews that discuss time perception in greater detail [5],[11],[15]–[18]. In the remaining sections below, we focus our discussion of time perception on the millisecond to second (referred to as sub-second) time scale and its relationship to activity in posterior parietal cortex.

Deficits in the perception of short time intervals are observed in individuals suffering from schizophrenia and other psychiatric conditions [19],[20]. Most of the motor actions we carry out on a daily basis require millisecond coordination between muscle contractions. Importantly, our conscious perception of external stimuli is linked to parietal areas that integrate information over these same time scales [21]. Thus, we constantly evaluate how our sensory inputs are changing many times a second, a time scale that is relevant for keeping track of our own movements [22]–[24] and those of external objects.

## Cortical Measurement of Sub-Second Time Intervals

**Figure 1:**
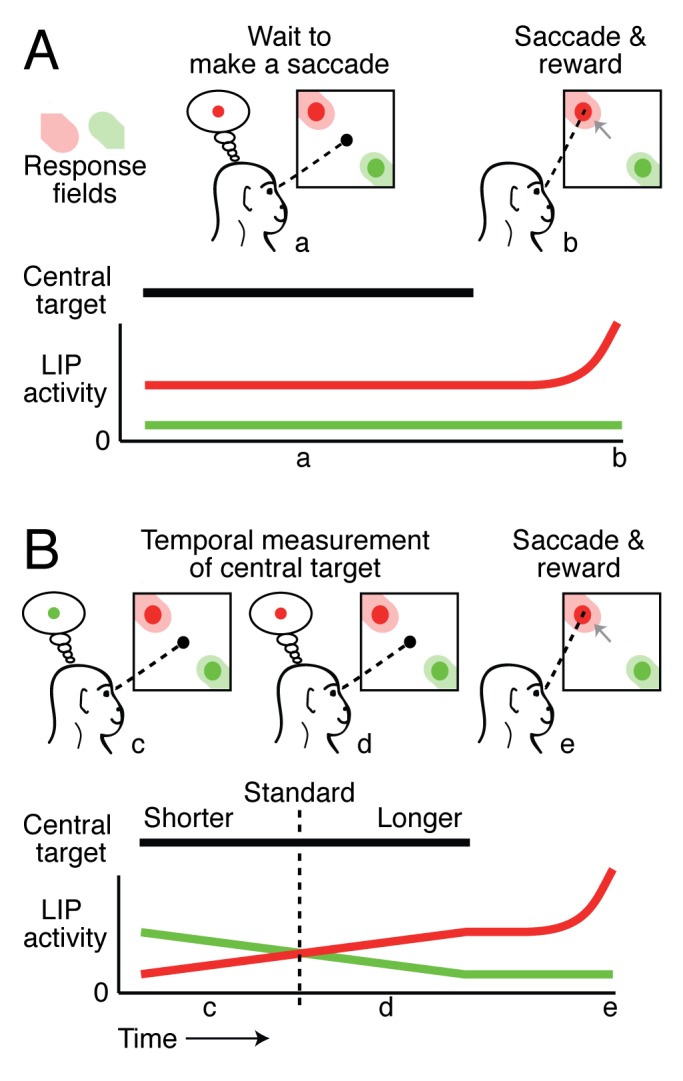
LIP activity during saccade and temporal measurement tasks. (A) Typical delayed saccade task. A monkey is trained to fixate (a) on a central target (black) while two peripheral targets are presented (red and green circles). Before the trial starts, the animal is cued to make a saccade (gray arrow) to the red target when the central target is extinguished (b). During the delay period (a) LIP neurons with response fields overlapping the saccade target (red trace) have sustained activity levels that are higher than LIP neurons with response fields located elsewhere (green trace). In addition, LIP neurons show an increased level of activity just before the saccade is made into their response field and a reward is received (b). (B) Temporal measurement task of Leon and Shadlen, 2003. Monkeys were trained to report the duration of a central target compared to a standard duration (dashed vertical line) by either a saccade to the green target to indicate a duration shorter than the standard or a saccade to the red target to indicate a longer duration. At the start of each trial, while the duration of the central target was still shorter than that of the standard, LIP responses preferentially reflected the location of the green target (c). As the trial progressed, however, the duration of the central target eventually exceeded the standard, and the monkeys' impending saccade was now directed to the red target (d). The activity of an LIP neuron with a response field corresponding to the short target (green trace) initially exhibited higher activity than a similar LIP neuron corresponding to the long target (red trace). As the trial progressed the activity levels of these two neurons reversed in a way that followed the monkey's judgment of the duration of the central target. An increase in LIP activity also occurred in this task just before the saccade (e).

Recently, a number of studies have examined potential single-neuron correlates of time perception in non-human primates [6],[25],[26]. By training the animals to indicate their perception of temporal duration and recording neural activity during performance of the task, these studies have consistently found correlates of time perception in the parietal cortex, specifically the lateral intraparietal area (LIP). Although LIP is active during many different types of behavioral functions, it has been known for many years to be specifically involved in the selection of targets for eye movements. Evidence for this role comes from classic studies [27], in which monkeys were trained to execute rapid eye movements (called saccades) to look at specific visual targets. In a standard experiment, the monkey is cued to saccade to a particular target (Figure 1A). The firing rate of a typical LIP neuron is elevated while the monkey waits to saccade (a) and then further increases just before the saccade (b), but only if the target is in a specific region of visual space known as the neuron's “response field.” Crucially, the monkey is typically given a reward for executing the correct saccade.

In an important study, Leon and Shadlen [6] exploited the role of LIP neurons in target selection to study the neural substrates of time perception. Specifically, they designed a task in which the monkey had to choose a saccade target based on its own measurement of elapsed time. This task is illustrated in Figure 1B, where the animal estimated the duration of a central target compared to a previously shown standard duration. If the animal's perception of the central target was shorter than that of the standard (c), it made a saccade to the green target; otherwise, it made a saccade to the red target (d). If the saccade was made to the correct target (e), the animal received a reward.

From behavioral measurements, Leon and Shadlen confirmed that monkeys were able to perform the time perception task and that their performance conformed to the pattern alluded to earlier, with discrimination being more sensitive (in terms of absolute time) for short intervals than for long ones. LIP activity generally encoded the direction of the saccade (Figure 1B), but it also had clear links to elapsed time. In particular, for long-duration trials, LIP activity increased steadily with the likelihood that a saccade into the response field would be required (d). These results demonstrated that in principle, it is possible to infer the duration of the central target based only on the firing rates of LIP neurons.

The clever approach implemented in the Leon and Shadlen experiment might be called a Temporal Measurement paradigm, as success in the task depends crucially on the animal's ability to measure the duration over which each stimulus is presented. Indeed in follow-up work [25], it was shown that LIP signals anticipate the timing of the occurrence of a salient event, specifically a visual stimulus cuing the execution of a saccade. In this regard, it seems that LIP contains neurons that are able to keep track of the time until a sensory event that is itself associated with a particular action.

The idea that LIP activity is involved in the timing of actions was also suggested by a 2006 study by Maimon and Assad [26], who showed that the responses of many LIP neurons signalled the arrival of a behaviourally relevant time point, even when disassociated from the motor response. This result suggests a more general role for LIP neurons in timing the execution of movements in response to external cues and supported the notion that LIP activity generally tracks the probability that a particular event is about to occur. Consequently, for events that can be anticipated in advance, a reasonable model of timekeeping could be based on steadily increasing LIP activity that reaches a threshold when an anticipated event is imminent.

## Parietal Cortex Activity During a Temporal Production Task

These previous studies suggest that LIP neurons provide signals related to the timing of external, behaviorally relevant events. But what about the situation in which the subject is required to monitor the passage of time in the absence of any anticipated external cue? This is arguably the more natural situation, but it seems rather difficult to examine in the laboratory, where experiments are typically designed around trials consisting of a fixed sequence of controlled events. For example, in the Leon and Shadlen paradigm, the presentation of the standard duration was always followed by the presentation of central target (Figure 1B), which was followed by a cue to move, the eye movement, and finally the reward.

**Figure 2:**
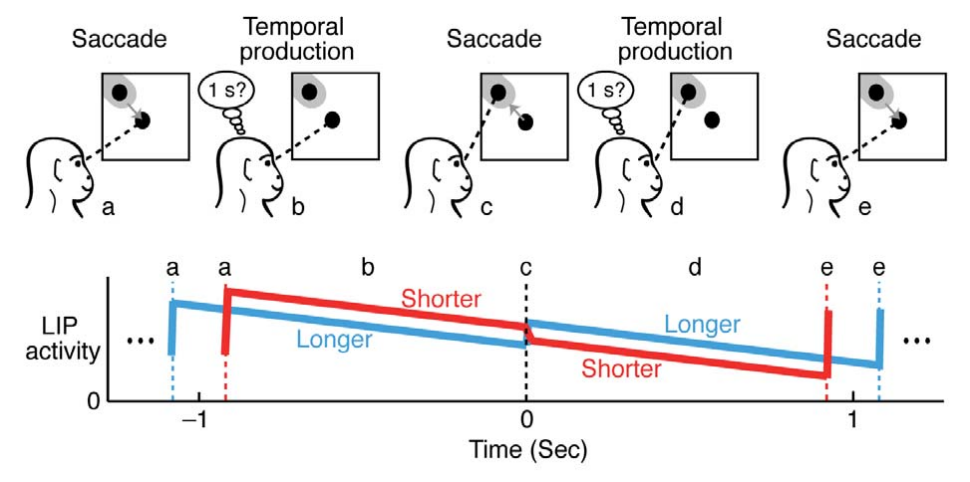
LIP activity during the temporal production task of Schneider and Ghose, 2012. Monkeys were trained to repetitively saccade (gray arrows) between a central and peripheral target at 1-s intervals. The monkeys were rewarded at a random time only if all the proceeding saccade intervals were within 200 ms of the 1,000 ms goal, and no sensory cue was provided to indicate the true interval duration. When the monkey made a saccade to the central target (a), LIP activity increased because the peripheral target was brought into the response field (gray region). While the animal waited to make a saccade back to the peripheral target (b), LIP activity slowly declined, but was greater for shorter saccade intervals (red trace) compared to longer intervals (blue trace). After the saccade to the peripheral target (c), the correlation between LIP activity and saccade interval reversed (d) until the next saccade to the central target (e). Interestingly, there was no pre-saccadic increase in LIP activity before the saccade to the peripheral target (c).

To minimize the potential influence of predictable external events, such as reward, Schneider and Ghose [1] devised a “Temporal Production” task (Figure 2), in which the subject must execute saccades rhythmically, in a back-and-forth manner. The timing of the saccades is left to the subject, but correct performance requires that each saccade be executed at a fixed time (in this case, 1 second) after the end of the previous one. The monkeys were rewarded for making saccades that were separated by temporal intervals of 800–1,200 ms. Crucially, the animals were unable to anticipate the arrival of the reward, as it was dispensed at random intervals during performance of the task. As in the studies mentioned above, neural recordings were performed in LIP.

The results were quite surprising for two reasons. First, during performance of the Temporal Production task, LIP activity actually *decreased* at a steady rate as the monkeys prepared to make saccades (Figure 2). This contrasts with the findings of previous studies that generally reported that LIP activity increased in anticipation of the externally cued movement. Indeed it differs from the majority of past LIP studies, which have classically reported that individual LIP neurons increase their firing rates in advance of a saccade toward their response fields.

A second surprising aspect of the temporal production task was the correlation between LIP activity and the animals' estimates of the 1-s duration. Schneider and Ghose found that small variations in LIP firing could predict similar variations in the animals' estimates of time and that the sign of this correlation *reversed* depending on the direction of the upcoming saccade (Figure 2; compare red versus blue LIP activity for short versus long interval estimates, respectively). These findings suggest that there is something about the Temporal Production task that profoundly changes the response patterns in LIP.

The authors suggest that a key difference between their task and many of those used previously is the predictability of the reward. Because the arrival of the reward is often tied to the end of the trial, it is possible that some observations of rising LIP activity reflected to some degree the anticipation of the reward, though this criticism does not apply to all previous observations of rising LIP activity (e.g., [25]). In the Temporal Production task, the timing of the reward was unpredictable, and the rising activity was not observed. This interpretation is plausible, given previous findings that responses in LIP are modulated by reward expectation [28].

To relate their LIP data to their behavioral results, Schneider and Ghose propose a “push-pull” model that relies on the fact that each brain actually contains two area LIPs (one in each cerebral hemisphere). Each LIP is generally more strongly activated for saccades directed to the opposite (contralateral) visual field, although there is some activity for saccades into the same (ipsilateral) visual field as well. If one assumes that time is measured as the difference between the activity in area LIPs contralateral and ipsilateral to the saccade direction, it becomes possible to recover a linear measure of time. A similar differencing operation has been proposed in many other contexts, including visual perception [29],[30] and decision making [31], so it seems likely to be a common aspect of brain function.

Intuitively the model proposed by Schneider and Ghose is akin to an hourglass that is flipped every time the sand runs out. In this analogy the steady release of the sand corresponds to declining LIP activity, and slight variations in the rate at which the sand falls account for variations in saccade timing. Extending the analogy a bit further, one can imagine the flip of the hourglass, corresponding to the completion of a saccade, as resetting the timer, erasing all memory of timekeeping over previous intervals. Schneider and Ghose identified a possible correlate of this resetting of the hourglass in a small up-down modulation of firing rate that occurs around the time of each saccade (Schneider and Ghose, Figure 5).

## Concluding Remarks

By studying neural responses during performance of a novel Temporal Production task, Schneider and Ghose have discovered LIP response dynamics that are significantly different than those previously reported. This by itself is no small feat, given that LIP is a well-studied brain region that is known for being involved in a large variety of different behavioral functions. What are the implications of these results?

First, although the push-pull model proposed by Schneider and Ghose accounts nicely for their results, it remains to be seen how well it generalizes to other experimental paradigms. In particular it will be interesting to see if it can account for previous observations of correlations between LIP activity and behavior, which appear to be highly sensitive to the task design.

Second, it is not clear why the LIP activity falls between saccades in the temporal production task. Is this a passive property of single-neuron firing, perhaps akin to adaptation? Or is it a neural code that emerges from the interactions of many neurons, and if so, is this code especially useful for keeping track of time?

Finally, and perhaps most importantly, to what extent can LIP activity be said to be causally involved in time perception? This relates to the question, raised at the beginning of this Primer, of the extent to which timekeeping in the brain is a local or a distributed process. One way to probe the role of LIP in time perception would be to artificially manipulate firing rates during the intersaccadic intervals and then to observe whether the animal's internal metric of time changed in a predictable way.

## Abbreviations

LIP: lateral intraparietal
